# Localized Overheating Phenomena and Optimization of Spark-Plasma Sintering Tooling Design

**DOI:** 10.3390/ma6072612

**Published:** 2013-06-25

**Authors:** Diletta Giuntini, Eugene A. Olevsky, Cristina Garcia-Cardona, Andrey L. Maximenko, Maria S. Yurlova, Christopher D. Haines, Darold G. Martin, Deepak Kapoor

**Affiliations:** 1 Department of Mechanical Engineering, College of Engineering, San Diego State University, 5500 Campanile Dr., San Diego, CA 92182, USA; E-Mails: diletta.giuntini@gmail.com (D.G.); cristina.cgarcia@gmail.com (C.G.-C.); 2 Key Laboratory for Electromagnetic Field Assisted Materials Processing, Engineering Physics University, Moscow 115409, Russia; E-Mails: azagorodnya@rambler.ru (A.L.M.); yurmary@mephist.ru (M.S.Y.); 3 US Army Armament Research, Development and Engineering Center (ARDEC), Picatinny Arsenal, NJ 07806, USA; E-Mails: christopher.d.haines2.civ@mail.mil (C.D.H.); darold.g.martin2.civ@mail.mil (D.G.M.); deepak.kapoor.civ@mail.mil (D.K.)

**Keywords:** Spark Plasma Sintering (SPS), Field Assisted Sintering (FAST), finite element, modeling, temperature distribution, overheating

## Abstract

The present paper shows the application of a three-dimensional coupled electrical, thermal, mechanical finite element macro-scale modeling framework of Spark Plasma Sintering (SPS) to an actual problem of SPS tooling overheating, encountered during SPS experimentation. The overheating phenomenon is analyzed by varying the geometry of the tooling that exhibits the problem, namely by modeling various tooling configurations involving sequences of disk-shape spacers with step-wise increasing radii. The analysis is conducted by means of finite element simulations, intended to obtain temperature spatial distributions in the graphite press-forms, including punches, dies, and spacers; to identify the temperature peaks and their respective timing, and to propose a more suitable SPS tooling configuration with the avoidance of the overheating as a final aim. Electric currents-based Joule heating, heat transfer, mechanical conditions, and densification are imbedded in the model, utilizing the finite-element software COMSOL™, which possesses a distinguishing ability of coupling multiple physics. Thereby the implementation of a finite element method applicable to a broad range of SPS procedures is carried out, together with the more specific optimization of the SPS tooling design when dealing with excessive heating phenomena.

## 1. Introduction

Spark Plasma Sintering (SPS) is a field-assisted powder consolidation technique showing a number of advantages over conventional powder processing methods. Higher heating rates allow shorter processing times and minimized grain growth; the rapid and volumetric heating (in case of electrically conductive powders) is simultaneous to the application of pressure, leading to highly dense structures; the pulsed DC current renders it suitable for a variety of materials; in many cases, the need for sintering aids is eliminated [1,2,3,4,5]. As a direct consequence, costs are lowered and the production of dense nano-materials, *i.e.*, endowed with improved mechanical properties, is possible.

Parallel to the attempts of simulating the SPS process by means of finite element simulations, several efforts have been made to develop a physical model capable to describe the involved underlying phenomena and the evolution of the process. Few constitutive models have been proposed, focusing on the influence of governing parameters of a thermal and non-thermal nature [6,7,8,9,10,11,12,13,14,15,16,17,18], while the need for reliable numerical simulation approaches was becoming gradually more important. Some studies could consider only uncoupled physical aspects of SPS [19,20,21,22,23,24,25,26,27,28], while, on the other hand, coupled modeling offers the opportunity of predicting the real outcomes of experimental procedures. During the recent years, a number of efforts have been dedicated to this objective. Finite element codes have been implemented, initially involving only parts of the physics characterizing the SPS, before the arrival of more complete approaches [29,30,31,32,33,34,35,36,37,38,39,40,41,42,43,44], comprehensive of the coupled phenomena constituting the process.

The first modeled aspects of the SPS process’ physics implemented in a coupled system were the electrical and the thermal phenomena [19,20,21,22,23,24], followed by the addition of mechanical models, equipped to simulate stresses and displacements in the specimens [29,30,31,32]. The most recent approaches include densification models [36,37,38] rendering the realization of a fully integrated electrical, thermal, mechanical, and constitutive finite element macro-scale model of spark plasma sintering.

These most recently developed approaches enable the direct analysis of the inter-related non-uniformities in current, temperature, and stress distributions, which can be more precisely modeled and controlled.

For the description of the SPS process, the understanding of the dynamic evolution of the process and material parameters is required. Zavaliangos *et al.* [19] have developed an Finite Element Method (FEM) model of SPS, which included the analysis of the temperature distribution in the SPS tooling. The conducted study of temperature distributions was intended to compare the temperature of the specimen with the temperature of the outer surface of the die, *i.e.*, the point where temperature measurements are generally obtained. An implementation of contact resistances between tooling components was also provided.

After the first works based on static analyses, some studies involving the implementation of the time evolution of material parameters, together with tracing the inhomogeneity of their spatial distributions, have been conducted [19,20,21,22,23,24,25]. Muñoz and Anselmi-Tamburini [29] established a discrete element model, which included such characteristics, focusing mainly on the specimen material inhomogeneities and on the non-uniformity of stress distributions. This and other modeling approaches considered simple-shape systems, such as a disk [28,29] or a rod-like part of the SPS setup [33,34].

In the present work, on the other hand, the modeling of the entire SPS tooling setup is indispensable, since the temperature distribution in the tooling is of principal interest. This study is inspired by a concrete problem of overheating in the SPS pressing setup observed in a number of experiments. Such phenomenon was found to happen regularly one or two minutes after the activation of the SPS device, and revealed itself in the top punch becoming red-hot—the occurrence that endangered the SPS equipment. Therefore, a macro-scale model of SPS is implemented, in order to investigate the evolution of temperature distributions within the SPS setup by varying the geometry of the SPS tooling, and to individuate the most effectual tooling configurations in lowering the peak temperatures.

In contrast to the above-mentioned study of Zavaliangos *et al.* [19], the present study is focused on the temperature distribution within the spacers and the punches, since the hottest point, as appeared from experiments, is located in that area. A finite element model developed based on the COMSOL™ platform for an SPS scalability study [35,36] has been considered and adapted to the considered overheating problem, therefore focusing the analysis on the tooling instead of the specimen; and underway, providing the utilized modeling framework with a more comprehensive description of the electric-thermal finite-element module.

## 2. Problem formulation

**Figure 1:**
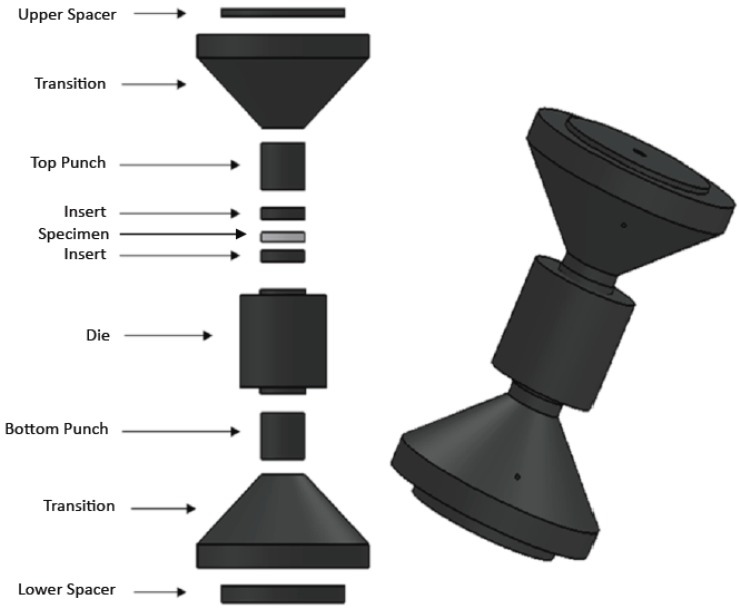
Spark Plasma Sintering (SPS) tooling manifesting the overheating problem.

The SPS press-form setup exhibiting the overheating problem during experimental procedures was a tooling typically employed in the SPS devices manufactured by FCT Co. (Figure 1). This tooling is characterized by a conical transition between punches and external spacer. More precisely, the considered setup involves an assembly of a die, two punches, two inserts separating the specimen and the punches, two conical (“tapered”) transitions, and two external spacers. The assembly components were made from Isocarb 85 graphite (obtained from Electrodes Inc., Milford, CT, USA). Dimensions of the tooling are reported in Table 1.

**Table 1:** Dimensions for Conventional 40 mm Tooling.

Component	Dimension	Value
Sample	Height [mm]	7.9
	Radius [mm]	20.0
Die	Height [mm]	80.0
	Radius [mm]	40.0
Punch	Height [mm]	40.0
Insert	Height [mm]	10.0
CC-Spacer	Height [mm]	20.0
	Radius [mm]	80.0
Transition	Height [mm]	80.0
	Radius 1 [mm] Radius 2 [mm]	20.0 80.0

**Figure 2:**
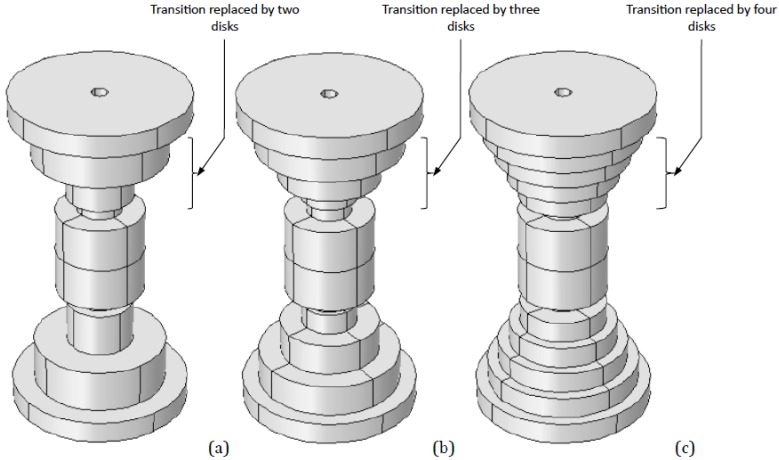
Geometries of (**a**) two transitional disks; (**b**) three transitional disks, and (**c**) four transitional disks configurations.

In our study, in order to decrease the temperatures reached during the SPS processes, the tapered configuration has been replaced by a variable number of “steps”, namely disks with different radii and thicknesses (this configuration based on a simple disk-like shape of the spacers also lowers the cost of tooling manufacturing). Several disks’ configurations have been designed, by varying their dimensions and number, for a total of 17 different setups (see Figure 2).

The following configurations have been modeled:

- Transition replaced by two disks:
  ○ Radii: 50 mm and 25 mm. Heights: 40 mm each.
  ○ Radii: 60 mm and 30 mm. Heights: 40 mm each.
  ○ Radii: 65 mm and 35 mm. Heights: 40 mm each.
  ○ Radii: 70 mm and 40 mm;
    ▪ Heights: 40 mm and 40 mm.
    ▪ Heights: 30 mm and 50 mm.
- Transition replaced by three disks:
  ○ Radii: 65 mm, 45 mm and 25 mm;
    ▪ Heights: 20 mm, 30 mm, and 30 mm.
    ▪ Heights: 30 mm, 20 mm, and 30 mm.
    ▪ Heights: 30 mm, 30 mm, and 20 mm.
  ○ Radii: 70 mm, 50 mm, and 30 mm;
    ▪ Heights: 20 mm, 30 mm, and 30 mm.
    ▪ Heights: 30 mm, 20 mm, and 30 mm.
    ▪ Heights: 30 mm, 30 mm, and 20 mm.
- Transition replaced by four disks:
  ○ Radii: 80 mm, 70 mm, 60 mm, and 50 mm;
    ▪ Heights: 20 mm each.
    ▪ Heights: 10 mm, 20 mm, 20 mm, and 30 mm.
  ○ Radii: 75 mm, 65 mm, 55 mm, and 45 mm. Heights: 20 mm each.
  ○ Radii: 70 mm, 60 mm, 50 mm, and 40 mm. Heights: 20 mm each.
  ○ Radii: 68 mm, 56 mm, 44 mm, and 32 mm. Heights: 20 mm each.
  ○ Radii: 66 mm, 54 mm, 42 mm, and 30 mm. Heights: 20 mm each.

The experimental procedure reproduced in FEM computations consisted in subjecting an alumina powder specimen, with initial density *ρ*_0_ = 65%, to a temperature regime composed by a sequence of 3.3 min of 400 K/min heating rate, followed by 10 min of holding at 1600 K, for a total of 810 s of the simulated time. The calculations correspond to an assumed conventional for SPS experimental setup when the temperature measurement was operated by the pyrometer, focused on the hole on the die surface. The processing is supposed to be conducted in temperature-control mode (not current-control), which means that the temperature profile is imposed by setting it on the SPS machine control panel

The macro-scale model of SPS enables the calculation of the evolution of voltage (*V*), temperature (*T*), stress (*σ*) and porosity (*θ*). From the latter, the relative density (*ρ*) can be immediately inferred as *ρ = 1 − θ*.

To simulate the system behavior, four components need to be combined: the electrical current density distribution, the consequent heat transfer by Joule heating, the sintering constitutive equation and the continuity equation for densification. As shown in [36], the equations that need to be implemented in the finite element macro-scale model can be defined as follows.

The DC current contribution is described as:

(1) $$-\nabla \cdot (\sigma_{el}\nabla V)=0$$

which is a result of the respective conservation equation, by expressing the electric field as the gradient of the electrostatic potential. Here, *σ_el_* is the electrical conductivity, given as a function of temperature.

Electrical current-related boundary conditions consist in the electrical insulation of the tooling outer surfaces, the grounding of the bottom surface of the lower ram, the application of the electrical potential at the top surface of the upper ram, and the possibility of choosing ideal or non-ideal (*i.e.*, including a resistance) behavior for the contact surfaces between the different tooling components. As an initial condition, the voltage was set to zero.

Coupled to Equation (1), the heat transfer due to Joule heating is modeled as:

(2) $$\rho_{eff}C_{p}\frac{\partial T}{\partial t}-\nabla \cdot (k_{T}\nabla T)=\sigma_{el}{\left|\nabla V\right|}^{2}$$

which is the equation for heat transfer by conduction, where the heat flux is expressed as −(*k_T_* ∇*T*) and the heat generated per unit volume by Joule effect, *σ_el_*|∇*V*|^2^, is the term that enables the coupling of electrical and thermal modules. The parameters given in functional form are *C_p_*, *k_T_* and *ρ_eff_*. *C_p_* is the heat capacity and *k_T_* is the thermal conductivity, both given as functions of temperature. Notice that the value of density included in the equation is:

(3) $$\rho_{eff}=\rho_{th}\rho =\rho_{th}(1-\theta )$$

*ρ_th_* indicates the theoretical density of the specimen’s material.

Heat transfer-related boundary conditions are given by heat loss due to heat radiation at the external surfaces (ambient temperature *T*_0_ = 300 K), by constant temperature (300 K) at the bottom surface of the lower ram and at the top surface of the upper ram, and by ideal contacts between layers.

Surface radiation is described as:

(4) $$-n\cdot \left(-k_{T}\nabla T\right)=\nu \varepsilon \left(T_{0}^{4}-T_{w}^{4}\right)$$

where the left-hand side is the heat flux per unit area, *ν* is surface emissivity (see Table 2), *ε* is the Stefan-Boltzmann constant, 5.6704 × 10^−8^ W/m^2^ K^4^, *T_w_* and *T*_0_ are the temperature of the external die surface and the ambient temperature, respectively. The initial temperature was fixed as 300 K.

Electrical and thermal properties are defined both for the graphite tooling and the alumina specimen. The mentioned parameters for Equations (1), (2) and (4) are given in Table 2 and Table 3.

Functional forms are used to describe the dependence of graphite properties on temperature and of alumina compact properties on temperature and porosity. Clearly, the model is intended to reproduce the dynamics of SPS processes, and both temperature and porosity are evolving during spark plasma sintering. A progressive tuning of these functions leads to a calibration of the material properties [36], aimed at reproducing the available experimental data.

The sintering constitutive equation is taken as follows [8]:

(5) $$\sigma_{ij}=\frac{\sigma (W)}{W}\left[\phi {\dot{\varepsilon }}_{ij}+\left(\psi -\frac{1}{3}\phi \right)\dot{e}\delta_{ij}\right]+P_{L}\delta_{ij}$$

where *σ_ij_* corresponds to the externally applied stress tensor components, ${\dot{\varepsilon }}_{ij}$ to the strain rate tensor components, *W* to the “equivalent strain rate”, *σ(W)* to the “equivalent stress”, which can be described by different expressions depending on the constitutive behavior of the powder compact, *ϕ* and *ψ* to the normalized shear and bulk viscosities, *P_L_* to the effective sintering stress and *δ_ij_* to the Kronecker delta.

**Table 2:** Graphite properties—*T*, *K*.

Parameter	Value	Units
Electrical conductivity, *σ_el_*	$\frac{3}{6.083\times 10^{-6}+1.4585\times 10^{-6}T/1000+2.3568\times 10^{-6}/(T/1000)}$	S/m
Thermal conductivity, *k_T_*	$\left\{ \begin{array}{l} -0.026\left(T-250\right)+60\\ 27.5 \end{array}\right.\;\begin{array}{l} T<1500\\ T\geq 1500 \end{array}$	W·(m·K)^−1^
Heat capacity, *C_p_*	$\left\{ \begin{array}{l} 4\left(T-280\right)+709.12\\ 8000 \end{array}\right.\;\begin{array}{l} T<2000\\ T\geq 2000 \end{array}$	J·(kg·K)^−1^
Density, *ρ*	1850	kg/m^3^
Emissivity, *ν*	0.8	–

**Table 3:** Alumina properties—*T*, *K*.

Parameter	Value	Units
Electrical conductivity, *σ_el_*	$\frac{(1-\theta )\times 10^{-8}}{1+2\theta }$	S/m
Thermal conductivity, *k_T_*	$\frac{6.51813304\times 10^{7}+T}{-6.696288\times 10^{5}+8.17585\times 10^{2}T}(1-1.5\theta -0.5\theta^{2})$	W·(m·K)^−1^
Heat capacity, *C_p_*	$\left(\frac{777.025T}{249.4+T}+\frac{790.15T}{249+T}+0.008T\right)\left(1-\theta \right)$	J·(kg·K)^−1^
Density, *ρ_eff_*	3970(1−θ)	kg/m^3^
Activation energy for self-diffusion, Δ*H_SD_*	$520\times 10^{3}$	J/mol
PLC parameter, *Ã*	5000	Pa·s^m^/°C^m^
Creep exponent, *m*	0.33	

The above-mentioned parameters are given as follows [8]:

(6) $$\phi ={\left(1-\theta \right)}^{2}$$

(7) $$\psi =\frac{2}{3}\frac{{\left(1-\theta \right)}^{3}}{\theta }$$

(8) $$P_{L}=\frac{3\alpha }{r_{0}}{\left(1-\theta \right)}^{2}$$

where *α* is the surface tension (1.12 N/m), *r*_0_ is the average initial particle radius (1 μm), $\dot{e}$ and $\dot{\gamma }$ are the first and second invariant of the strain rate tensor and strain rate tensor deviator, respectively.

The porosity evolution is defined through the continuity equation, from which the progressive densification of the powder compact can be derived:

(9) $$\frac{\dot{\theta }}{1-\theta }=\dot{e}$$

Initial porosity is assumed to be uniform throughout the specimen and equal to 35%.

Expressions (10) and (11) describe the nonlinear viscous constitutive law (power-law creep) used to model the porous material behavior under elevated temperatures and to predict sintering deformation. The effective stress has the form:

(10) $$\sigma (W)=AW^{m}$$

Here, the equivalent strain rate is taken as:

(11) $$W=\frac{1}{\sqrt{1-\theta }}\sqrt{\phi {\dot{\gamma }}^{2}+\psi {\dot{e}}^{2}}$$

and the material constant *A* is expressed by means of an Arrhenius-type relationship [36]:

(12) $$A=\tilde{A}T^{m}\exp\left(\frac{m\Delta H_{SD}}{RT}\right)$$

where *Ã* and *m* are power-law creep parameters, and *ΔH_SD_* is the activation energy for self-diffusion. The corresponding power-law creep parameters for alumina are reported in Table 3. The creep exponent *m* is considered constant, while in reality it may vary with temperature. For our purposes this approximation does not cause significant alterations of the results. Nevertheless, a deeper investigation of finite element methods intended to implement the power-law creep mechanisms could lead to considerable improvements in SPS simulations.

Concerning mechanical boundary conditions, the surface of the specimen in contact with the lower punch is fixed, the specimen lateral movement is impeded in the radial direction in order to simulate the confinement in a rigid die, and the top surface of the sample is subjected to an externally applied pressure.

As previously mentioned with regards to electrical boundary conditions, for the same 17 configurations an electrical contact resistance between the different parts of the setup has been incorporated. Simulations have been executed for both cases: with and without contact resistance, in order to evaluate the impact of this parameter.

In a real setup the resistance depends on the size and the shape of the tooling, on the properties of the materials in contact and on the contact surface area. Furthermore, as the SPS setup is usually subjected to a uniaxial (vertical) pressure, the contacts on horizontal planes tend to be tighter than the contacts in vertical planes. Therefore electrical conductivities are higher in the horizontal contacts than in the vertical contacts. Table 4 displays the electrical conductivities used for the calculations, whose values are taken from [39]. In general, contact resistances change during sintering, because of the change in the contact surface area.

**Table 4:** Parameters used for Gap Electric Conductivity in the Contact between Parts.

Gap Electric Conductance Graphite—Graphite			Gap Electric Conductance Graphite—Specimen		
Horizontal Contact	$\sigma_{gg,h}$	1.25 × 10^5^ S/m^2^	Horizontal Contact	$\sigma_{gs,h}$	1.25 × 10^4^ S/m^2^
Vertical Contact	$\sigma_{gg,v}$	7.5 × 10^4^ S/m^2^	Vertical Contact	$\sigma_{gs,v}$	7.5 × 10^3^ S/m^2^

A distinction is made also based on the materials in contact: electrical resistance for contacts between the same material (such as graphite-graphite, *gg*) is lower than the one between different materials (graphite-powder specimen, *gs*), thus *gg* electrical conductivity is higher than *gs* conductivity. An arbitrary decreasing factor for conductivity is used for the contact between the graphite tooling and the specimen.

**Figure 3:**
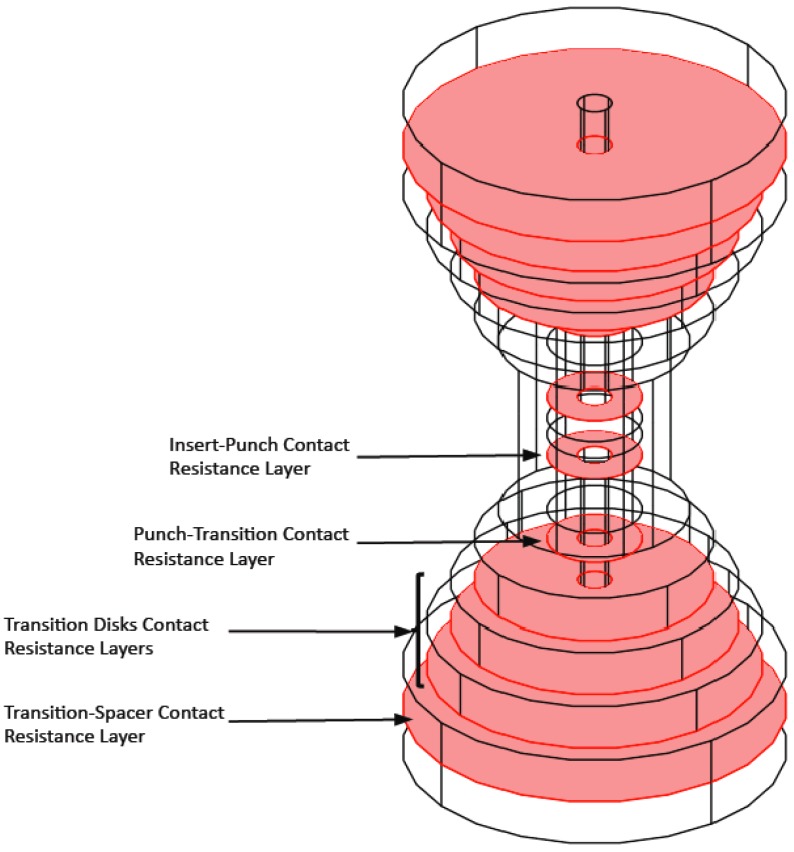
Graphite-graphite horizontal contact resistance layers highlighted for one of the four transitional disks configurations.

As an example, Figure 3 highlights the graphite-graphite contact area for the horizontal case in a four disks setup. The other setups have analogous contact areas.

In the conducted computations the set of contact resistances is simulated as a thin layer of arbitrary thickness between the two contact surfaces [39], implemented in COMSOL™ by means of distributed impedance in between the disks composing the transition. The equation for the distributed impedance is:

(13) $$n\cdot \left(J_{1}-J_{2}\right)=\sigma_{g}V$$

where $\left(J_{1}-J_{2}\right)$ is the difference in current density between the two contact surfaces and $\sigma_{g}$ takes the value of $\sigma_{gg,h}$, $\sigma_{gg,v}$, $\sigma_{gs,h}$ or $\sigma_{gs,v}$, depending on the case considered.

The finite element software employed in the present study is COMSOL™ 4.2, thanks to its capability of solving multiple coupled physics simultaneously. This software enables the implementation of the four mechanisms indicated by Equations (1), (2), (5) and (9), in parallel with the materials models described by Equations (10) and (12). Different physics were coupled by employing electrical currents, heat transfer, solid mechanics, and PDE modules, while substituting the default parameters with the calibrated features, which are provided in Table 1, Table 2 and Table 3.

The information on voltage and external pressure was taken from the available experimental data [35]. By imposing the applied voltage in the FE implementation, the temperature profiles obtained with the SPS equipment were reproduced, with minor discrepancies, negligible in the present study, due to the fact that in experimental procedures involving the SPS there is typically no direct control over the voltage, but only on the temperature regime experienced by the specimen (through controlling the electric current).

Regarding the finite-element mesh, various attempts have been performed in order to obtain reliable and stable numerically converging results. The free tetrahedral mesh with the number of elements varying for different considered cases in the range 16,000–22,000 generated by the COMSOL™ software, with a size classified as “normal”, “fine”, or smaller, revealed to be the most effective in conducting the 3D-FE calculations.

## 3. Results and Discussion

**Figure 4:**
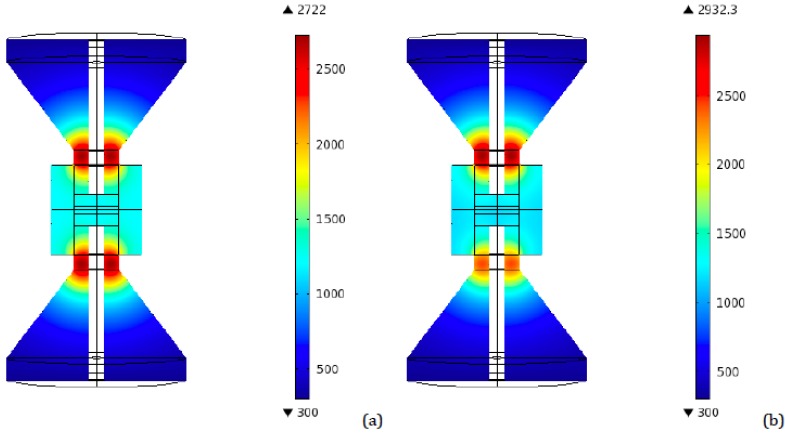
Conventional SPS 40 mm tooling—Peak temperatures (K) distribution for (**a**) Ideal contact; (**b**) Resistive contact.

As a preliminary step, a simulation was conducted for the 40 mm diameter tooling configuration, in order to have a reference set of data. Figure 4 represents the highest temperatures distribution in an axial plane for the conventional 40 mm tooling, for both ideal and resistive contact. The peak in temperatures occurs after 90 s from the beginning of the process, which is in agreement with previous works utilizing finite element simulations, even if conducted with different software [21].

### 3.1. Ideal Contacts

A first set of calculations is conducted for the different tooling setups without including electrical contact resistances.

The temperature distribution in the vertical central plane obtained at the end of the prescribed temperature regime for the two, three, and four disks configurations have been considered. The highest value, attained at the punch, is close to 1922 K and corresponds to the four disks configurations with the largest disks radii.

**Figure 5:**
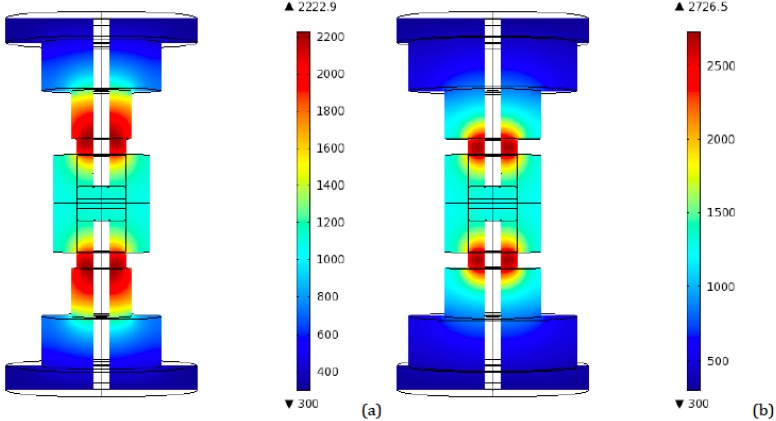
Peak temperature distribution (K)—Ideal contact—two transitional disks configuration. (**a**) Radii: 50 and 25 mm—Heights: 40 mm each; (**b**) Radii: 70 and 40 mm—Heights: 40 mm each.

**Figure 6:**
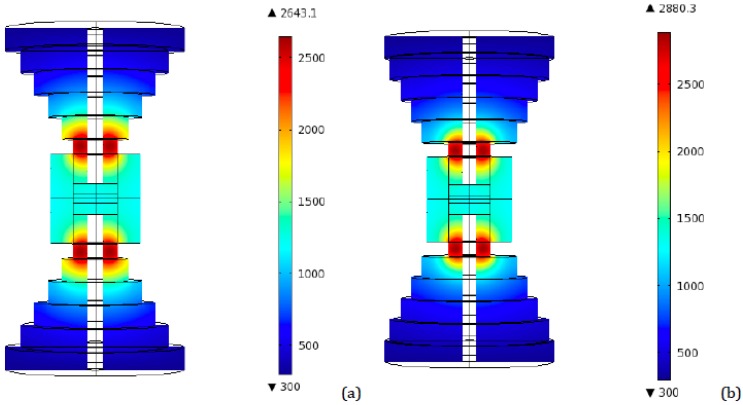
Peak temperature distribution (K)—Ideal contact—four transitional disks configuration. (**a**) Radii: 66, 54, 42, and 30 mm—Heights: 20 mm each; (**b**) Radii: 75, 65, 55, and 45 mm—Heights: 20 mm each.

More interestingly, the temperature distribution in the vertical central plane corresponding to the time interval between 90 and 100 s (depending on the particular configuration), for the time steps at which temperatures reach their highest values, was monitored. Figure 5 and Figure 6 show the resulting plots for the two and four disks setups, from which the direct identification of the point of the punch, in which the peak temperature is attained, is possible. Here it can be noticed that the highest value, reached again at the punch, is close to 2917 K and corresponds to the same four disks configuration in which the highest final temperature is reached (radii: 80, 70, 60, and 50 mm). This is a remarkable result, since this peak is even greater than the one obtained for the conventional tapered tooling (2722 K), indicating that an increase in the number of disks and their diameters is not the correct way of solving the problem of overheating. The same happened for final (regime) temperatures: this same four disks configuration has a regime temperature of 1922 K, while the conventional tool reaches 1827 K.

On the other hand, it may be noticed that the temperature distribution is symmetrical, thus the temperature along the disks is equivalent for top and bottom modified transitions.

The configuration in which the heat penetrates more in depth of the transitional disks corresponds to the two disks setup, where a temperature of 1200 K is found at 20 mm from the punch. In this same area the temperatures in the other configurations are approximately 1000 K for the four disks configuration and 1100 K for the three disks configuration.

The time evolution of temperature during the simulations is evaluated too. Figure 9a,c,e, shows the temperature *vs.* time plots during the whole time interval of 810 s, for two, three, and four disks respectively. The temperatures shown are a volume average in the top punch. The volume average is calculated as the integral over the volume, divided by the volume itself. From these graphs the time step corresponding to the peak can be inferred, and the range in between 90 and 100 s is confirmed.

For all the configurations considered, in terms of number of disks, a decrease in the radii values leads to a decrease in the overall temperatures.

### 3.2. Resistive Contacts

To provide our simulations with a more accurate reproduction of real experimental conditions, an electrical contact resistance between graphite-graphite contact areas, as well as graphite-specimen contact areas, is introduced, as described previously. For comparison purposes, the same boundary conditions applied in the ideal contact case are employed. Specifically, the voltage utilized to produce the above-mentioned heating regime for the ideal contact case is used in this context too.

Due to the differences in the electrical configuration of the system, distinct results are obtained. In particular, although the highest temperature is still attained at the punch, the temperature distribution is no longer symmetric in the axial planes: once the electrical contact resistance is introduced, dissimilarities in temperatures between top and bottom punches appear. Temperatures in the top punch tend to be higher, while the heat experiences more difficulties in reaching the specimen.

**Figure 7:**
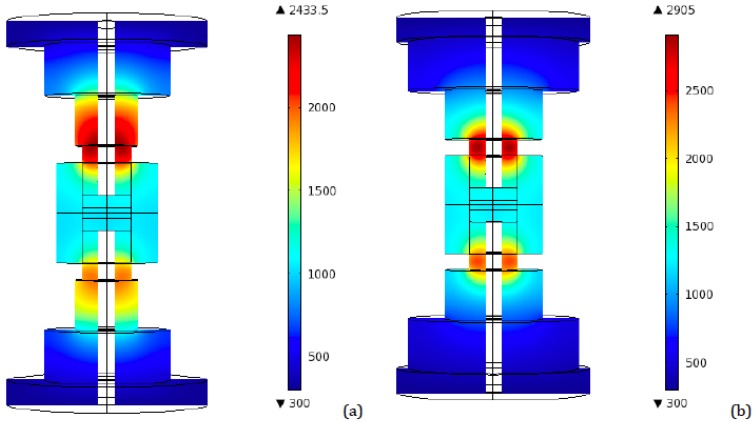
Peak temperature distribution (K)—Resistive contact—two transitional disks configuration. (**a**) Radii: 50 and 25 mm—Heights: 40 mm each; (**b**) Radii: 70 and 40 mm—Heights: 40 mm each.

**Figure 8:**
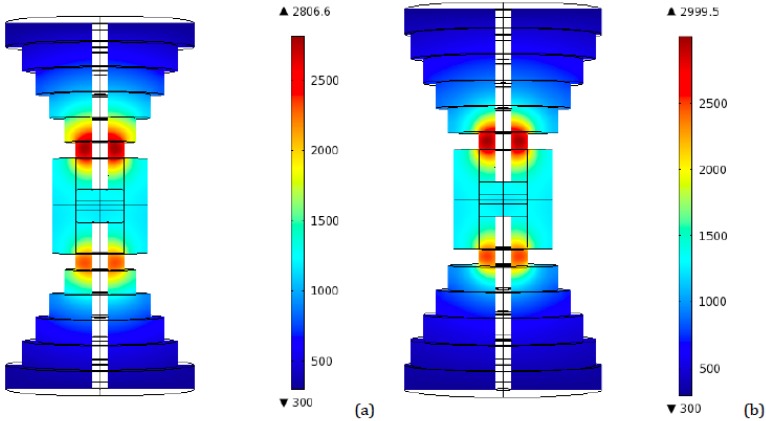
Peak temperature distribution (K)—Resistive contact—four transitional disks configuration. (**a**) Radii: 66, 54, 42, and 30 mm—Heights: 20 mm each; (**b**) Radii: 75, 65, 55, and 45 mm—Heights: 20 mm each.

**Figure 9:**
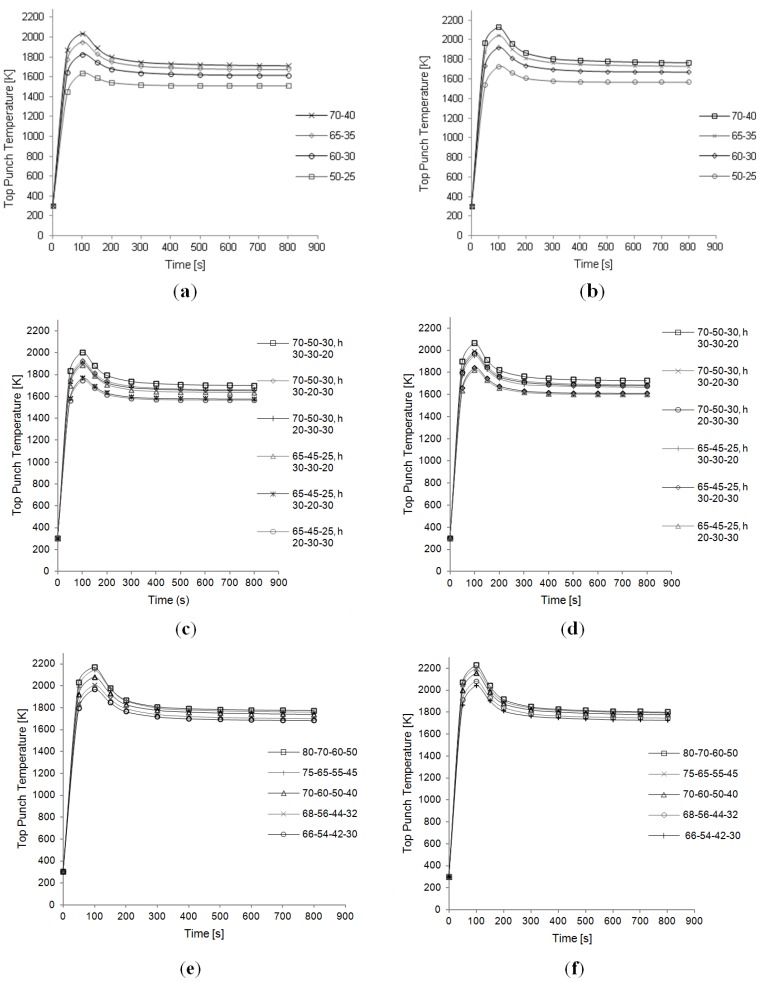
Temperature evolution in the top punch. (**a**) two disks setup—Ideal contact; (**b**) two transitional disks setup—Resistive contact; (**c**) three transitional disks setup—Ideal contact; (**d**) three transitional disks setup—Resistive contact; (**e**) four transitional disks setup—Ideal contact; (**f**) four transitional disks setup—Resistive contact. Dimensions of the setup are listed in the legend. The first three values correspond to disks radii, from top to bottom; the last three values (marked by the letter “h”) indicate the respective heights.

Plots analogous to the ones produced for the ideal contact case are provided in Figure 7, Figure 8 and Figure 9.

In Figure 7 and Figure 8 the temperature distribution in the axial plane corresponding to the 90–100 s time interval is shown, *i.e.*, the peak temperatures are displayed, for two and four disks setups, respectively. In Figure 9b,d,f the temperature evolution with time (volume average in the top punch) for the three configurations, varying the geometry, is shown.

Concerning the location of the peak and the role of the disks radii, analogous considerations to the ideal contact case can be inferred.

### 3.3. Discussion

**Figure 10:**
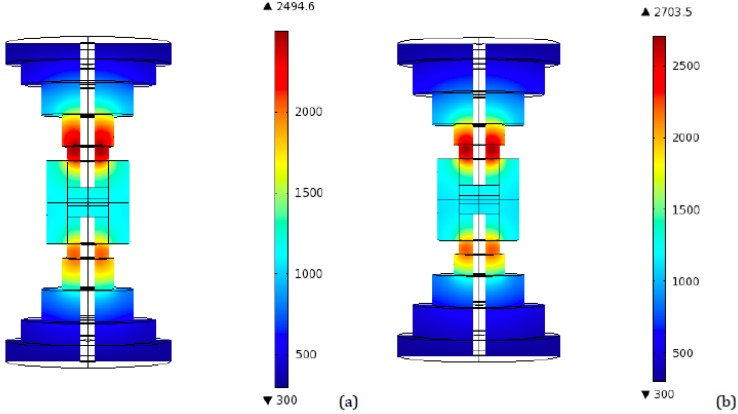
Peak temperature distribution—Resistive contact—Height influence comparison for a three disks configuration (Radii: 65, 45, and 25 mm). (**a**) Heights: 20, 30, and 30 mm from the top; (**b**) Heights: 30, 30, and 20 mm from the top.

From the temperature plots obtained (see Figure 10) one can see that an increase of the height of the disk adjacent to the top punch, while decreasing accordingly the height of the top disk (the one with the largest radius), causes a diminution of the global temperature. A reason for this behavior may be attributed to the larger surface area exposed to the environment (at *T*_0_ = 300 K) close to the hottest zone, which allows for a more efficient surface radiation (see boundary conditions for the thermal module).

**Figure 11:**
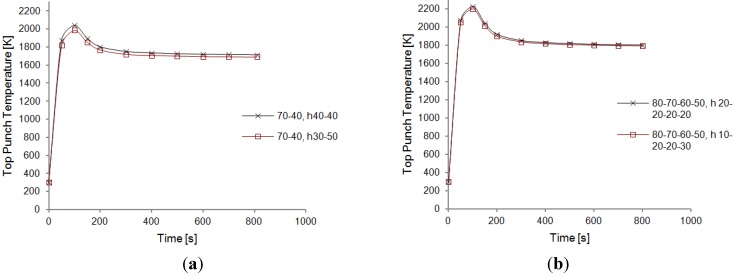
Temperature evolution—Effect of height variation. (**a**) two transitional disks—Ideal contact—Radii 70 and 40 mm—Height 40 mm each compared with 30 and 50 mm; (**b**) four transitional disks—Resistive contact—Radii 80, 70, 60, and 50 mm—Height 20 mm each compared with 10, 20, 20, and 30 mm. Dimensions of the setup are listed in the legend. The first three values correspond to disks radii, from top to bottom; the last three values (marked by the letter “h”) indicate the respective heights.

With this result in mind, the same procedure was attempted for the two disks and four disks configurations. In both cases, the setups with the largest radii were chosen. In the two disks case, ideal contact was simulated, while for the four disks configuration the resistive contact model was run. As represented in Figure 10 and Figure 11, again, an increase in the bottom disk height, at the expenses of the top disk one, leads to a decrease in temperatures, even if not substantial. The effect of this geometrical modification appears to be more significant in the ideal contact case than in the resistive contact one.

The differences between ideal contact and resistive contact are made clear by comparing Figure 5 and Figure 7, Figure 6 and Figure 8, and by observing the graphs represented in Figure 9. Temperatures reach higher values in the resistive case. The highest peaks of overheating are reached for the four disks configuration, which, in fact, is the one characterized by the largest number of interfaces provided with a “resistive layer”.

Notice again that in several setups the temperatures are even higher than the ones obtained in the conventional tapered tool, as pointed out in the ideal contact case, too. In particular, the final and peak temperatures are greater than the conventional tool ones (1934 and 2932 K, respectively), for all the four disks configurations with radii greater or equal to the 70, 60, 50 and 40 mm combination.

Moreover, to verify the impact that changes in electric contact resistance can have in the temperature distribution, a different arrangement of contact conductivities has been simulated in a specific case, *i.e.*, the two disks setup (radii: 60 and 30 mm, heights 40 mm each). Here, only horizontal electric contact resistances were considered and the configuration of the graphite-specimen contact is changed to obtain a larger resistivity; these modifications lead to a considerable increase in the top punch temperature (2020 K, instead of 1853 K).

Some remarkable data are obtained from the comparison of the temperatures of the “hot point” of the top punch with the temperatures attained in the specimen (for which a volume average is computed). The key point in the presently applied approach for the analysis of the overheating problem is based exactly on these derived data, distinct from previous works (e.g., [19]), in which the calculated temperature difference was, instead, the one between the specimen and the external surface of the die, that is at the point where SPS temperatures are measured.

**Figure 12:**
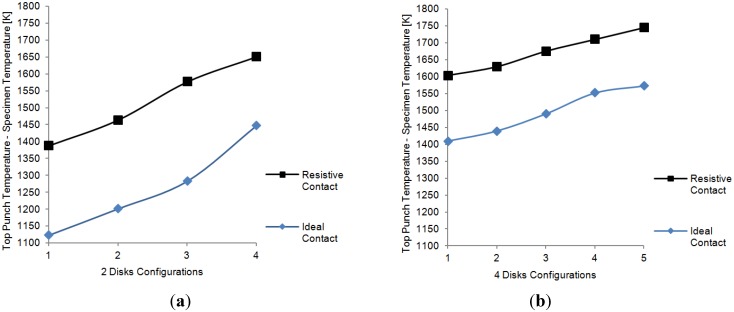
Comparison of maximum (transient) temperature difference between top punch and specimen. (**a**) two transitional disks configurations. Radii: (1) 50 and 25 mm; (2) 60 and 30 mm; (3) 65 and 35 mm; (4) 70 and 40 mm; (**b**) four transitional disks configurations—Radii: (1) 66, 54, 42, and 30 mm, (2) 68, 56, 44, and 32 mm, (3) 70, 60, 50 and 40 mm, (4) 75, 65, 55, and 45 mm; (5) 80, 70, 60, and 50 mm.

Figure 12 compares these two temperatures for the two and four disks cases, respectively, (a) and (b), with the different radii combinations along the *x*-axis and the peak and specimen temperatures difference along the *y*-axis. In both cases all the disks had the same height. The maximum temperature difference corresponds to the peak of the transient temperature and is inferred from the maximum temperature distribution plots. In all the cases, the temperature of the punch for the resistive contact case is higher than the temperature of the punch for the ideal contact. An opposite situation is seen at the specimen, where a slightly higher temperature is obtained in the ideal contact case. Although the presence of the resistive contact reduces the efficiency of the system, the temperature difference obtained in the specimen between the ideal contact case and the resistive case is relatively small. The case where the difference is the largest (four disks configuration), which seems to suggest a less efficient system, is precisely the one that is able to heat the specimen more (~1600 K). Therefore, the energy losses on the setups seem to depend more on the configuration of the disks than on the actual characteristics of the contacts, at least for the combination of material and electrical contact resistance properties used in the simulations.

In a similar way, the final temperature difference between the top punch and the specimen for the four configurations, with ideal as well as resistive contacts, was investigated. Again, higher differences were obtained for the electric resistive contact case. In this stationary configuration, the differences in temperature vary with the radius of disks in an inversely proportional fashion and stay almost constant with height variations.

Once all the previous outcomes have been examined, it is evident that, in order to choose the most advisable setup, separate considerations can be made for every configuration investigated (categorized in terms of the number of disks).

One of the best choices is certainly the two disks configuration, since here the contact resistance is minimized to only one extra “resistive layer” with respect to the tapered setup. The lower the radii and the higher the bottom disk, the better results we get in terms of temperatures (e.g., radii of 50 and 25 mm, or, if these values are too small, since the bottom disk is almost as large as the punches, 60 and 30 mm, preferably with heights of 30 and 50 mm, respectively). In this case a peak temperature decrease of 500 K can be attained.

If the main interest is, instead, in having a configuration as similar as possible to the original conical (tapered) one, the four disks setup can be chosen. Again, the lower the radii and the higher the bottom disks, the better results are attained. Concerning our simulations, the best combination appears to be the one with radii 66, 54, 42, and 30 mm with heights equal to 10, 20, 20, and 30 mm, respectively. Lower values of the radii can be attempted to get even better results. It should be noted that in this case the most significant decreases in peak temperatures that can be obtained are in a range of 150–250 K.

A compromise solution between the minimum contact resistance and the tapered configuration is the three disks setup. The same considerations concerning radii and heights need to be applied. From our data, the configuration with radii 65, 45, and 25 mm, and respective heights of 20, 30, and 30 mm, appears to be a good option. The diameter of the top disk could be further decreased. The temperatures can be lowered by about 350 K.

It is important to notice that the efficiency of all the suggested configurations needs to be verified in terms of the specimen densification. The lowest peak temperatures correspond to lower temperatures inside the specimen, especially when the contact resistance is imbedded. Simulations and experiments need to be conducted in order to be sure that the specimen's porosity decreases significantly in reasonable time intervals. Additionally, other technological aspects, such as feasibility and easiness of production processes, should be taken into account.

## 4. Conclusions

An FEM model is developed in order to study an overheating problem detected in SPS tooling during SPS experimental procedures. The peak in temperatures occurred at the top punch and within a time interval well-identified by calculations.

By means of modifications to the original tapered geometry of the SPS tooling, several methods to lower tooling temperatures were identified. It emerged that the configuration in which the heat penetrates more in depth of SPS tooling is the two disks configuration, while the highest temperatures correspond to the four disks configurations with the largest disks radii, for both peak and intermediate time intervals. An increase in the number of disks can lead to even greater temperatures than the ones reached in the tapered tooling configuration case.

The calculations showed that properly chosen sets of cylindrical spacers are effective in decreasing the temperatures with respect to the single spacer configuration with conical transition. In addition, the manufacturing of cylindrical spacers is simpler than the manufacturing of conical-shape spacers; sets of cylindrical spacers provide also the adjustability of the tooling for the fabrication of specimens of different sizes.

Concerning the spacer transition dimensions, the following conclusions can be drawn: a decrease in the disks radius values causes a diminution of the global temperature; the same effect is achieved with the increase of the height of the disk adjacent to the top punch, accompanied by the consequent reduction of the height of the top disk, which is adjacent to the spacer.

The implementation of a resistive contact layer between the spacer disks produces higher temperatures as well as an additional obstacle to heat penetration towards the specimen, and provides an asymmetrical temperature distribution in the axial plane.

From an application standpoint, one of the most significant results is the comparison of the peak temperature in the tooling and the specimen temperatures. The disparity between these two values is higher when assuming the resistive contacts and is directly proportional to the disks radii.

In view of these outcomes, an advisable configuration could be the two spacer disks setup with the aforementioned geometrical considerations, since the extra resistive layers are minimized and the attainable decrease in peak temperatures reaches 500 K. The four spacer disks configuration can be selected if the aim is a minor alteration of the conical spacer geometry, although the maximum reduction obtainable is in a range of 150–200 K. The intermediate solution provided by three spacer disks can be opportunely tuned by modifying the disks height values, in addition to the radii. A solution needs to be selected based on the densification capabilities and on the additional technological and economic factors (operational convenience, cost of tooling manufacturing, *etc*.).
